# C/EBP alpha agonist ICCB280 overcomes doxorubicin resistance in osteosarcoma through mitochondrial dynamics-dependent GREM1-MAPK activation

**DOI:** 10.1038/s41419-026-08980-y

**Published:** 2026-06-25

**Authors:** Yuchen Han, Hui Li, Chunyuan Xue, Lu Pan, Li Liu, Hui Chen, Zhendong Wang, Fengkun Ji, Yinglong Zhang, Xiaofeng Kang, Ying Lu, Yimeng Du, Jianxiong Li, Wenchao Li

**Affiliations:** 1 Senior Department of Pediatrics, The Seventh Medical Center, Chinese PLA General Hospital, Beijing, China; 2 Medical School of Chinese PLA, Beijing, China; 3 Senior Department of Oncology, The Fifth Medical Center, Chinese PLA General Hospital, Beijing, China; 4 National Key Laboratory of Advanced Biotechnology, Academy of Military Medical Sciences, Beijing, China; 5 Senior Department of Orthopedics, The Fourth Medical Center, Chinese PLA General Hospital, Beijing, China

**Keywords:** Apoptosis, Cancer metabolism

## Abstract

The resistance of doxorubicin (DOX), the first-line chemotherapeutic drug for osteosarcoma (OS), stands as a pivotal obstacle in the effective treatment of OS. Alterations in mitochondrial dynamics and thereby affecting reactive oxygen species (ROS) accumulation play critical roles in DOX-induced cell death. However, the novel targets and pharmacological agents combating DOX resistance in OS via mitochondrial homeostasis and DOX-induced cell death manipulation is poorly understood. Herein, we showed that the C/EBPα/GREM1/p-ERK signaling pathway sensitizes OS to DOX by promoting mitochondrial fission and causing ROS-induced apoptosis. C/EBPα agonist ICCB280 and ERK pathway inhibitor PD98059 both played a DOX sensitivity augmentation role in OS in vitro and in vivo. The combined application of these two agents synergistically amplifies the cytotoxic impact of DOX, potentially overcoming DOX resistance and offering innovative therapeutic strategies for treating DOX-resistant OS.

**Figure:**
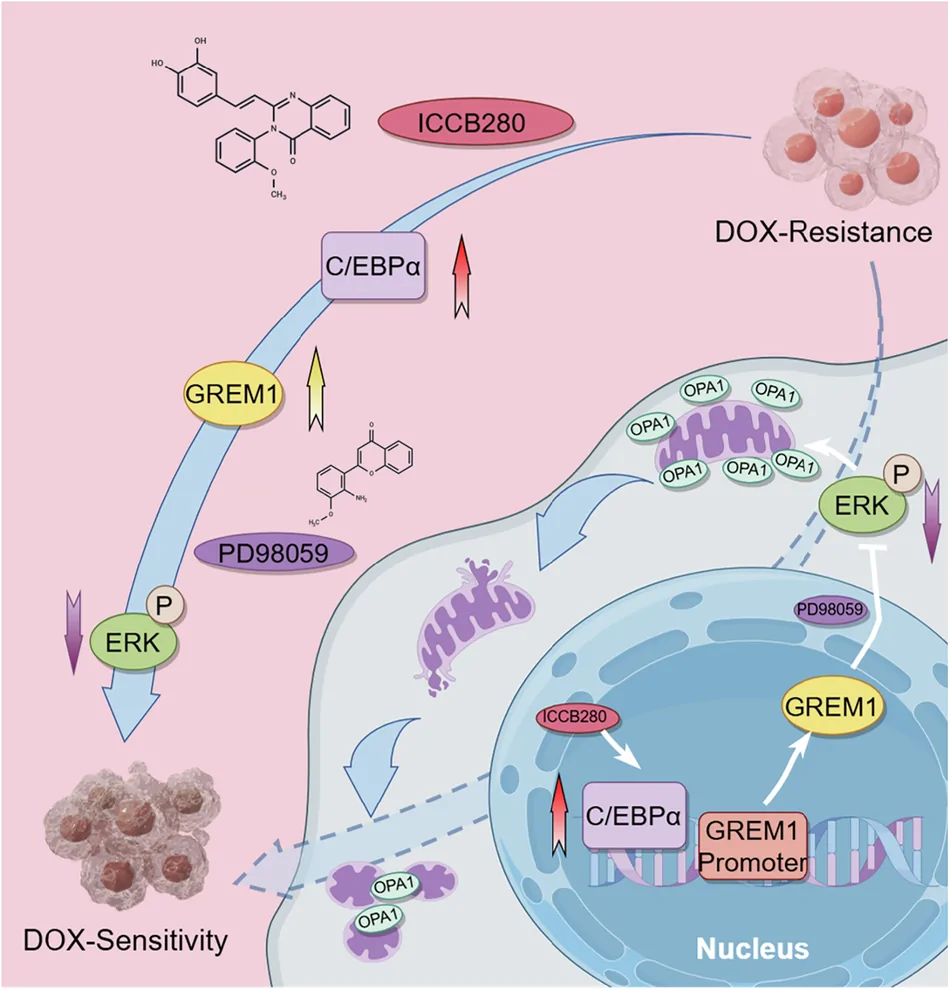
**SCHEME： ICCB280 and PD98059 co‑administration counteracts doxorubicin resistance in osteosarcoma.** When ICCB280 and PD98059 are co-administered, the activation of GREM1 by C/EBPα and the inhibition of ERK phosphorylation are intensified. This suppresses OPA1 expression, promotes mitochondrial fission, and effectively counteracts chemotherapy resistance in osteosarcoma.

**SCHEME： ICCB280 and PD98059 co‑administration counteracts doxorubicin resistance in osteosarcoma.** When ICCB280 and PD98059 are co-administered, the activation of GREM1 by C/EBPα and the inhibition of ERK phosphorylation are intensified. This suppresses OPA1 expression, promotes mitochondrial fission, and effectively counteracts chemotherapy resistance in osteosarcoma.

## Introduction

Osteosarcoma is the most common primary malignant bone tumor, primarily affecting children and adolescents. It exhibits a strong tendency for lung metastasis, and the mortality rate rises substantially once metastasis occurs [1]. The use of neoadjuvant chemotherapy has increased the 5-year overall survival rate in osteosarcoma patients from below 20% to over 60%. However, for those with recurrence or metastasis, the prognosis remains poor, with a 5-year survival rate below 25% [2]. Chemotherapy resistance continues to be a major barrier to successful osteosarcoma treatment. As such, identifying new therapeutic targets to counter resistance and developing more effective treatment strategies are of critical clinical relevance.

Mitochondria serve as the cells’ energy generators [3]. These highly responsive organelles can swiftly adjust to external stimuli and drug-induced stress via fusion and fission events [4]. Such mitochondrial dynamics help shape and distribute mitochondria under therapeutic pressure, supporting tumor cell survival and resistance to drugs [5]. Reactive oxygen species (ROS), largely derived from mitochondria, can cause oxidative stress and trigger cell death when excessively produced. The balance between mitochondrial homeostasis and ROS levels plays a central role in determining cell fate [6]. Still, how mitochondrial dynamics relate to chemoresistance in osteosarcoma remains insufficiently defined.

Doxorubicin (DOX), a first-line chemotherapeutic agent for osteosarcoma, induces cytotoxicity by forming DOX-DNA adducts, inhibiting topoisomerase, and causing DNA strand breaks [7–9]. Crucially, DOX also binds to cardiolipin in the inner mitochondrial membrane, leading to excessive ROS production, structural disruption of mitochondria, and ultimately cell death. Several studies have reported drugs that influence DOX sensitivity. For instance, AZD7648 increased sensitivity to DOX by blocking the PRKDC-linked PI3K/AKT pathway [10], and Cinobufagin raised DOX sensitivity by promoting FOXO1-driven transcription of FCGBP in osteosarcoma [11]. Yet, effective approaches that exploit the mitochondrial toxicity of DOX remain underexplored. Therefore, we aimed to screen for new targets and compounds that affect mitochondrial dynamics and provoke elevated ROS, rendering osteosarcoma cells to be more vulnerable to DOX and reversing resistance.

In this study, we examined gene expression differences between chemotherapy-sensitive and -resistant patients using the GEO database, identifying GREM1 as a gene strongly associated with chemosensitivity. Inhibiting GREM1 appeared to suppress DOX-induced apoptosis in osteosarcoma cells, likely through altered mitochondrial dynamics and function. We described how GREM1 regulates mitochondrial network fragmentation via OPA1 in an ERK1/2-dependent manner. Furthermore, we discovered that DOX reduces GREM1 expression primarily by suppressing the transcription factor CCAAT/enhancer binding protein α (C/EBPα). The cytotoxic effect of DOX can be intensified in vitro and in vivo by applying the C/EBPα agonist ICCB280 along with the ERK pathway inhibitor PD98059. This work identifies the C/EBPα/GREM1/p-ERK axis as a central regulator of DOX resistance, and the compounds identified in this context may serve as potential therapeutic options for DOX-resistant osteosarcoma.

## Materials and methods

### Plasmids, shRNA, and lentiviruses

The PCR-amplified coding sequence of GREM1 was inserted into the pCMV3-C-FLAG vector (Sino Biological, Beijing, China; #CV012). The GREM1 promoter and its mutated variants were cloned into the pGL3.0 Basic vector (Promega, WI, USA; #E1715). The GREM1-targeting shRNA sequence was: sense, 5’-GCAGTGTCGTTGCATATCCAT-3’; antisense, 5’-ATGGATATGCAACGACACTGC-3’. This shRNA construct was cloned into the pLKO.1 vector (MIAOLING BIOLOGY, Wuhan, China; #DB00024). Lentiviral particles were generated by co-transfecting the target plasmid with the packaging vectors psPAX2 and pMD2.G into 293 T cells. After 48 h, the viral supernatant was collected.

### Cell culture and treatment

Human osteosarcoma cell lines (MG63, U2OS, HOS) and the normal osteoblast cell line hFOB1.19 were obtained from Procell Life Science and Technology (Wuhan, China). The 143B osteosarcoma cell line and 293 T human embryonic kidney cells were sourced from Zhong Qiao Xin Zhou Biotechnology (Shanghai, China). All cells were maintained in DMEM with 10% fetal bovine serum (FBS, Gibco, NY, USA; #12100046) and penicillin-streptomycin (Gibco; #10099-141 C). While hFOB1.19 cells were cultured at 34 °C, all other lines were incubated at 37 °C in 5% CO_2_.

### Western blotting (WB)

Total protein was extracted using RIPA buffer with protease inhibitors. Samples were separated by gel electrophoresis, transferred to nitrocellulose (NC) membranes, and blocked. Membranes were incubated overnight at 4 °C with primary antibodies (listed below), followed by incubation with secondary antibodies for 1 h after TBS-Tween washes. Detection was carried out using the ChemiDoc Touch imaging system and ECL reagents. The following antibodies were used: GREM1 (Santa Cruz Biotechnology, CA, USA; #sc-515877), HGF (Proteintech, Wuhan, China; #26881-1-AP), C/EBPα (ZEN-BIOSCIENCE, Chengdu, China; #220693), Phospho-Akt (Ser473) (CST, MA, USA; #4060), AKT (CST; #9272), Phospho-p44/42 MAPK (Erk1/2) (Thr202/Tyr204) (CST; #4370), p44/42 MAPK (Erk1/2) (CST; #4695), TOMM20 (Proteintech; #11802-1-AP), MYC tag (Proteintech; #16286-1-AP), FLAG tag (Proteintech; #66008-4-Ig), OPA1 (ZEN-BIOSCIENCE; #382025), and HRP-conjugated secondary antibodies against rabbit and mouse IgG (ABMART, Shanghai, China; M21003).

### RNA sequencing (RNA-Seq)

Total RNA was extracted from U2OS cells for quality assessment, library construction, and sequencing. After sequencing, the data underwent comparative expression analysis, quantification, significance testing, and functional enrichment. Sequencing was performed using the Illumina NovaSeq 6000 platform.

### RNA extraction and real-time quantitative PCR (qRT-PCR)

Total RNA was extracted from cultured cells using TRIzol reagent (Invitrogen, CA, USA; #16096020). After RNA purification, cDNA was synthesized through reverse transcription. QRT-PCR was performed using TB Green® Premium Ex Taq II (Takara, Kyoto, Japan; #RR820), with β-actin used for normalization. The primer sequences were: GREM1, forward: 5ʹ-CGGAGCGCAAATACCTGAAG-3ʹ; reverse: 5ʹ-GGTTGATGATGTGC

GACTGT-3ʹ. HGF, forward: 5ʹ-GCTATCGGGGTAAAGACCTACA-3ʹ; reverse: 5ʹ-CGTAGCGTACCTCTGGATTGC-3ʹ. C/EBPα, forward: 5ʹ-CAAGAACAGCAACGA

GTACCG-3ʹ; reverse: 5ʹ-GTCACTGGTCAACTCCAGCAC-3ʹ. ERK, forward: 5ʹ- TACACCAACCTCTCGTACATCG-3ʹ; reverse: 5ʹ-CATGTCTGAAGCGCAGTAAG

ATT-3ʹ.

### Analysis of cell vitality

Cells were seeded into 96-well plates at a density of 2 × 10^3^ cells per well. After overnight incubation, drugs were added at specific concentrations and co-incubated with cells for 36 h. Cell viability was assessed using 10% Cell Count Kit-8 (CCK-8) (DOJINDO, Kumamoto, Japan; #CK04) for 2 h. Absorbance at 450 nm was measured using a Multiskan FC microplate photometer (Thermo Fisher Scientific, MA, USA). The compounds used were: Doxorubicin (Selleck; #E2516; C_27_H_29_NO_11_; Purity: 99.71%), ICCB280 (TargetMol, MA, USA; #T8839; C_23_H_18_N_2_O_4_; Purity: 98.04%), PD98059 (TargetMol; #T2623; C_16_H_13_NO_3_; Purity: 99.66%); Cisplatin (Selleck, TX, USA; #S1166; Cl_2_H_6_N_2_Pt; Purity: 99.84%); Ifosfamide (Selleck; #S1302; C_7_H_15_Cl_2_N_2_O_2_P; Purity: 99.24%); Methotrexate (Selleck; #S1210; C_20_H_22_N_8_O_5_; Purity: 99.79%).

### Colony formation assay

A total of 2 × 10^3^ cells were seeded into each well of a 6-well plate and cultured in 10% FBS-containing medium for 10 days. Cells were fixed with 4% paraformaldehyde for 30 minutes and stained with 1% crystal violet for another 30 minutes. Colony numbers were quantified using ImageJ software.

### Oxygen consumption rate (OCR) analysis

OCR was measured using the Seahorse XF Cell Mitochondria Stress Test Kit (Agilent Technologies, CA, USA; #103015-100) on the Seahorse XFe 96 Extracellular Flux Analyzer (Seahorse Bioscience, MA, USA). According to the manufacturer’s protocol, 10,000 cells were seeded per well in Seahorse XF 96 cell culture plates. Baseline readings were recorded before sequential injections of oligomycin, FCCP (p-trifluoromethoxy carbonyl cyanide phenylhydrazone), and the mitochondrial complex I inhibitor rotenone together with the complex III inhibitor antimycin A (Rote/AA). Data were analyzed using the Seahorse XF-96 Wave software. OCR values are expressed in pmol/min.

### Mitochondrial morphology assay

Cells (2 × 10^4^ cells/well) were plated on coverslips and incubated overnight. Mitochondria were stained in live cells with Mitotracker Green FM (Thermo Fisher Scientific; #M7514). Fluorescence images were acquired with a Nikon microscope system. Mitochondrial branch length was quantified using ImageJ software. The specific steps include: converting the MitoTracker fluorescence image into a binary image, using the “Skeleton” and “Analyze Skeleton” functions to obtain the skeletal information of the mitochondrial network, and calculating the average branch length (μ m).

### Transmission electron microscope (TEM)

After cell collection and fixation, samples were processed for resin embedding and sectioned. Ultrastructural analysis was performed using a HITACHI HT7700 transmission electron microscope operated at 80 kV.

### ROS and mitochondrial membrane potential (MMP) measurement

Cells were incubated with 2’,7’-Dichlorofluorescin diacetate (DCFH-DA) probes (Solarbio Life Sciences, Beijing, China; #D6470) to assess ROS levels or with JC-10 assay kits (Solarbio Life Sciences; #CA1310) to evaluate mitochondrial membrane potential. Flow cytometry data were analyzed using Flow Jo software.

### Analysis of apoptosis

Apoptosis in treated cells was measured using the Annexin V-FITC Apoptosis Detection Kit (Beyotime Biotechnology, Shanghai, China; #C1062). The percentage of apoptotic cells was quantified by flow cytometry, and data were processed with Flow Jo software. Additionally, Caspase-3/7 activation was assessed using the Caspase-3/7 Activity Assay Kit (Beyotime Biotechnology; #C1168).

### Cell transfection

Small interfering RNAs (siRNAs) targeting GREM1 and HGF (si-GREM1, si-HGF) were synthesized by JTS Scientific. si-ERK (CST; 6560) purchased from Cell Signaling Technology, si-C/EBPα (Santa Cruz Biotechnology; sc-37048) purchased from Santa Cruz Biotechnology. Transfection was performed using RNAIMAX reagent (Thermo Fisher Scientific; #13778030) when cells reached approximately 70% confluence, following the manufacturer’s protocol. The siRNA sequences were: GREM1, sense: 5ʹ-AAAUCGAUGGAUAUGCAACGA-3ʹ; antisense: 5ʹ-GUUGCAUAUCCAUCGAUUUGG-3ʹ. HGF, sense: 5ʹ-GCACACCAAUGUGCUAAUATT-3ʹ; antisense: 5ʹ-UAUUAGCACAUUGGUCUGCUGCTT-3ʹ.

### Immunohistochemical (IHC) detection

Tissue sections were deparaffinized in xylene and rehydrated through graded ethanol. Antigen retrieval was performed with 10 mM citric acid buffer. Sections were treated with 3% hydrogen peroxide and blocked with goat serum for 30 minutes to reduce non-specific binding. The slides were then incubated overnight at 4 °C with the primary antibody GREM1 (Santa Cruz Biotechnology; #sc-515877), followed by 1 h of incubation with a secondary antibody (ZSGB Bio, Beijing, China; #PV-6000) at room temperature. Staining was developed using 3,3ʹ-diaminobenzidine (DAB; ZSGB Bio; #ZLI-9017). All tissue samples were evaluated independently by three pathologists blinded to clinical information.

### Histological Immunofluorescent detection

After dewaxing, antigen retrieval, and serum blocking, primary antibodies GREM1 (Santa Cruz Biotechnology; #sc-515877), C/EBPα (ZEN-BIOSCIENCE; #220693), and P-ERK (CST; #4370) were applied to tissue sections, followed by incubation with secondary antibodies iF488 Tyramide (Servicebio, Wuhan, China; #G1231), iF546 Tyramide (Servicebio; #G1251), and iF440 Tyramide (Servicebio; #G1250). Serum blocking was repeated before each antibody incubation step. Nuclei were stained with DAPI, and slides were sealed for fluorescence scanning using the 3DHISTECH platform.

### Luciferase reporter assay

Wild-type and mutated GREM1 promoter sequences were cloned into the pGL3.0 basic vector. U2OS cells were seeded at 1 × 10^5^ cells per well in 24-well plates and incubated for 24 h at 37 °C prior to transfection. Cells were co-transfected with 1 μg of pGL3-GREM1-wt, pGL3-GREM1-mut, or pGL3-empty vector using Lipofectamine 3000 (Invitrogen; #L3000015). After 48 h, luciferase activity was measured according to the manufacturer’s instructions.

### Chromatin immunoprecipitation (ChIP) assay

ChIP analysis was performed using the Magna ChIP G assay kit (Millipore, MA, USA; #17-611). Chromatin was sheared by sonication and immunoprecipitated with an anti-C/EBPα antibody (Abcam, Cambridge, UK; #ab317442), followed by binding to protein G magnetic beads. The immunoprecipitated DNA fragments, including the GREM1 promoter and upstream regions, were amplified using Power SYBR Green PCR Master Mix (Applied Biosystems™, MA, USA; #4368577). Data were normalized to values from IgG and empty vector controls. The primer sequences used for qRT-PCR were: GREM1 Promoter, Forward: 5’-TACAAACTGTGGCTCACTG-3’; Reverse: 5’-GATACTTACGAAACACCCG-3’; GREM1 Upstream, Forward: 5’-CAGAAAACCCCTCTCGCCC-3’; Reverse: 5’-CGCTGGGAAGAGCGAGATC-3’.

### Paraffin tissue TUNEL staining

Apoptosis in tissue sections was evaluated using the TUNEL assay kit (Servicebio; #G1501). According to the manufacturer’s instructions, sections were first treated with Proteinase K working solution, followed by incubation with TdT reaction buffer at the recommended ratio. Nuclei were then stained with DAPI for 8 minutes.

### Immunofuorescence (IF)

Cells were fixed with 4% paraformaldehyde and permeabilized using 0.5% Triton X-100 on ice. Primary antibodies were applied and incubated overnight at 4 °C, followed by secondary antibody incubation at room temperature for 1 h. Nuclei were counterstained with DAPI, and samples were observed under a fluorescence microscope.

### Construction of nude mouse tumor model

This study received approval from the Ethics Review Committee of the General Hospital of the People’s Liberation Army of China (Approval No.: 2024KY0112-KS001). All procedures followed institutional guidelines and regulatory standards.The 6-week-old female nude mice were purchased from SPF Biotechnology. Subcutaneous Xenograft Model:Tumor cells were subcutaneously implanted in each experimental group (10-12 mice per group). Drug administration was initiated when a palpable, slightly raised mass (approximately 200 mm³ in volume) was detected. Tumor length and width were measured every 3 days, and tumor volume was calculated using the formula: Volume = (length × width²)/2. We strictly adhere to the standard tumor measurement procedures. When measuring with a vernier caliper, “length” is defined as the maximum diameter of the tumor mass, and “width” is defined as the maximum diameter perpendicular to the long diameter. All measurements of tumor bearing mice were carried out by two fixed technicians who were not familiar with the specific grouping of the experiment (single blind). Lung Metastasis Model: Tumor cells were injected via the tail vein in each experimental group (3 mice per group). Drug treatment was administered 3 weeks post-injection. Lung tumor growth was evaluated 6 weeks after tumor cell inoculation.

### Human clinical samples

A human osteosarcoma tissue microarray containing 70 osteosarcoma and 1 normal bone tissue sample was obtained from Zhongke Guanghua Intelligent Biotechnology Co., Ltd. (ZK bioaitech, Xi’an, China; #L714901). Additionally, 9 osteosarcoma tissue samples were collected from the PLA General Hospital. All patients provided written informed consent prior to sample collection, and the study was reviewed and approved by the Ethics Review Committee of the General Hospital of the Chinese People’s Liberation Army.

### Statistical analysis

Statistical analysis was carried out using GraphPad Prism 9.0 or SPSS 23.0. Data are shown as mean ± standard deviation (SD). Comparisons between groups were conducted using Student’s t-test. A significance threshold of *p* < 0.05 was applied. Each experiment included a minimum of three biological replicates.

## Results

### GREM1 identified as a critical gene for chemotherapy sensitivity in osteosarcoma

**Fig. 1:**
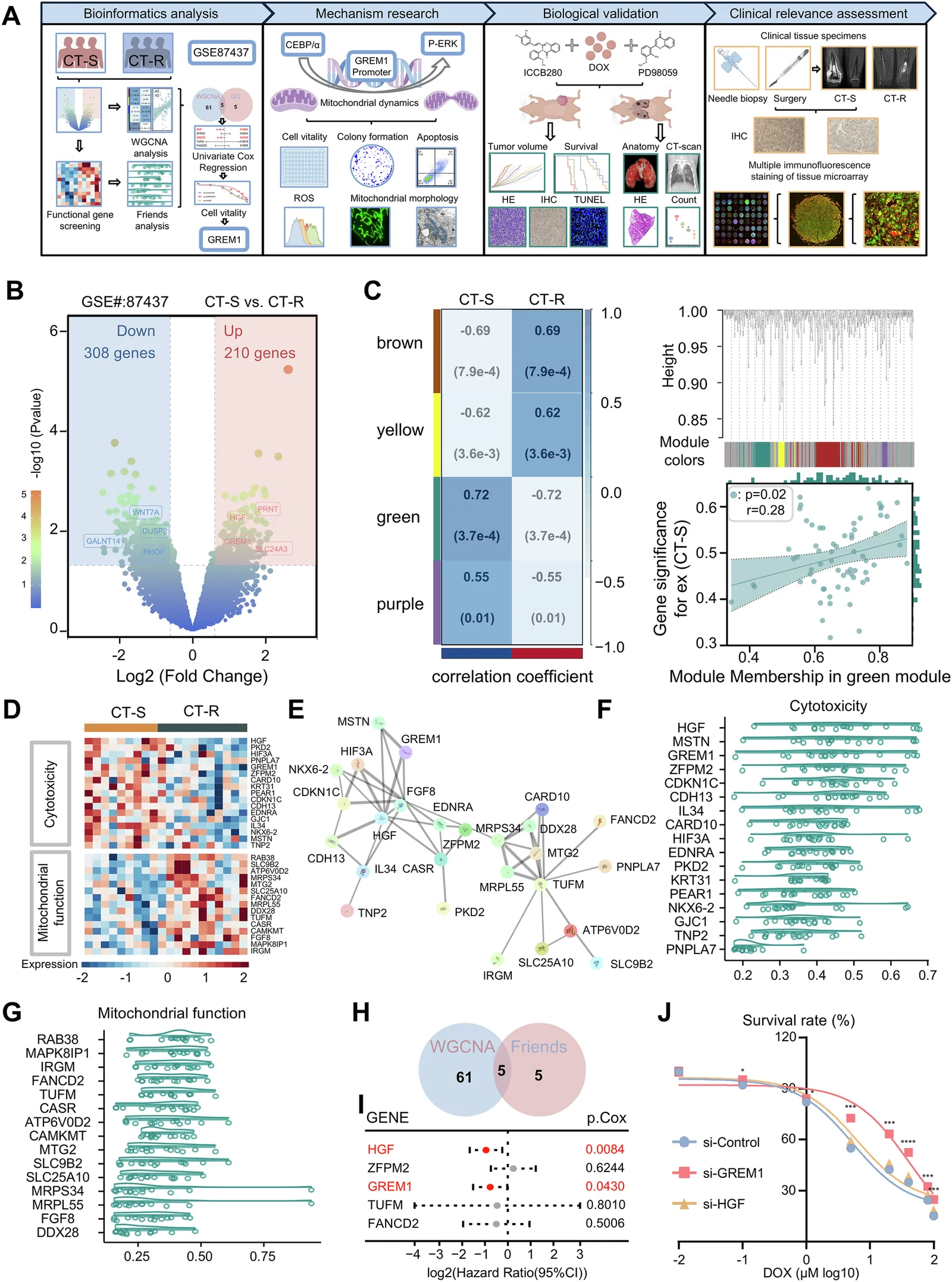
GREM1 identified as a critical gene for chemotherapy sensitivity in osteosarcoma. **A** Diagram of the study design used in this work. **B** Volcano plot showing differentially expressed genes (DEGs) between chemotherapy-sensitive (CT-S) and chemotherapy-resistant (CT-R) osteosarcoma patients from dataset GSE87437. **C** Four gene co-expression modules generated by WGCNA, with 66 genes identified in the green module. **D** Gene sets related to mitochondrial function and cytotoxicity among the DEGs. **E** Protein–protein interaction (PPI) network displaying gene interactions. **F**, **G** Friends analysis to explore inter-gene relationships. **H** Venn diagram identifying overlapping genes between selected sets. **I** Univariate Cox regression using prognostic data from the TARGET database. **J** CCK-8 assay assessing cell viability. *n* = 3. Error bars represent SD; *p* values calculated by two-tailed unpaired t-test; *p < 0.05; ***p < 0.001; ****p < 0.0001.

To explore new genes that may counteract DOX resistance in osteosarcoma, we used bioinformatics approaches to identify candidate therapeutic targets, as outlined in Fig. 1A. Based on the GEO dataset (GSE87437), patients were grouped into chemosensitive (CT-S) and chemoresistant (CT-R) categories using the Huvos classification. A total of 518 differentially expressed genes (DEGs) were identified between these groups, with 210 genes upregulated and 308 downregulated (Fig. 1B). To pinpoint gene clusters relevant to chemotherapy response, we performed weighted correlation network analysis (WGCNA) using the full gene expression profile and associated clinical data. Four gene modules emerged, with the green module showing the strongest association with chemotherapy sensitivity (Fig. 1C). Our analysis further revealed that the CT-R group displayed more active mitochondrial function and lower cytotoxicity compared to the CT-S group (Fig. 1D). We also noted intricate interactions between cytotoxic genes and those linked to mitochondrial function (Fig. 1E). From two functional modules, we selected the top five highest-scoring genes (Fig. 1F and G) and combined these with WGCNA-identified genes to search for new candidates that connect chemotherapy response to mitochondrial activity and cytotoxicity (Fig. 1H). We then carried out univariate Cox regression to evaluate the prognostic relevance of the five overlapping genes. Two of them—GREM1 and HGF—showed significant associations with overall survival in osteosarcoma patients (Fig. 1I). To test their functional impact, we knocked down each gene in osteosarcoma cell lines and assessed changes in DOX sensitivity (Supplementary Fig. S1A). The results indicated that GREM1 knockdown clearly increased resistance to DOX, while HGF knockdown did not have a noticeable effect (Fig. 1J). Thus, GREM1 emerged as a gene likely to influence chemotherapy sensitivity in osteosarcoma and was selected for further study.

### GREM1 enhances osteosarcoma sensitivity to DOX by promoting ROS-induced cell apoptosis

**Fig. 2:**
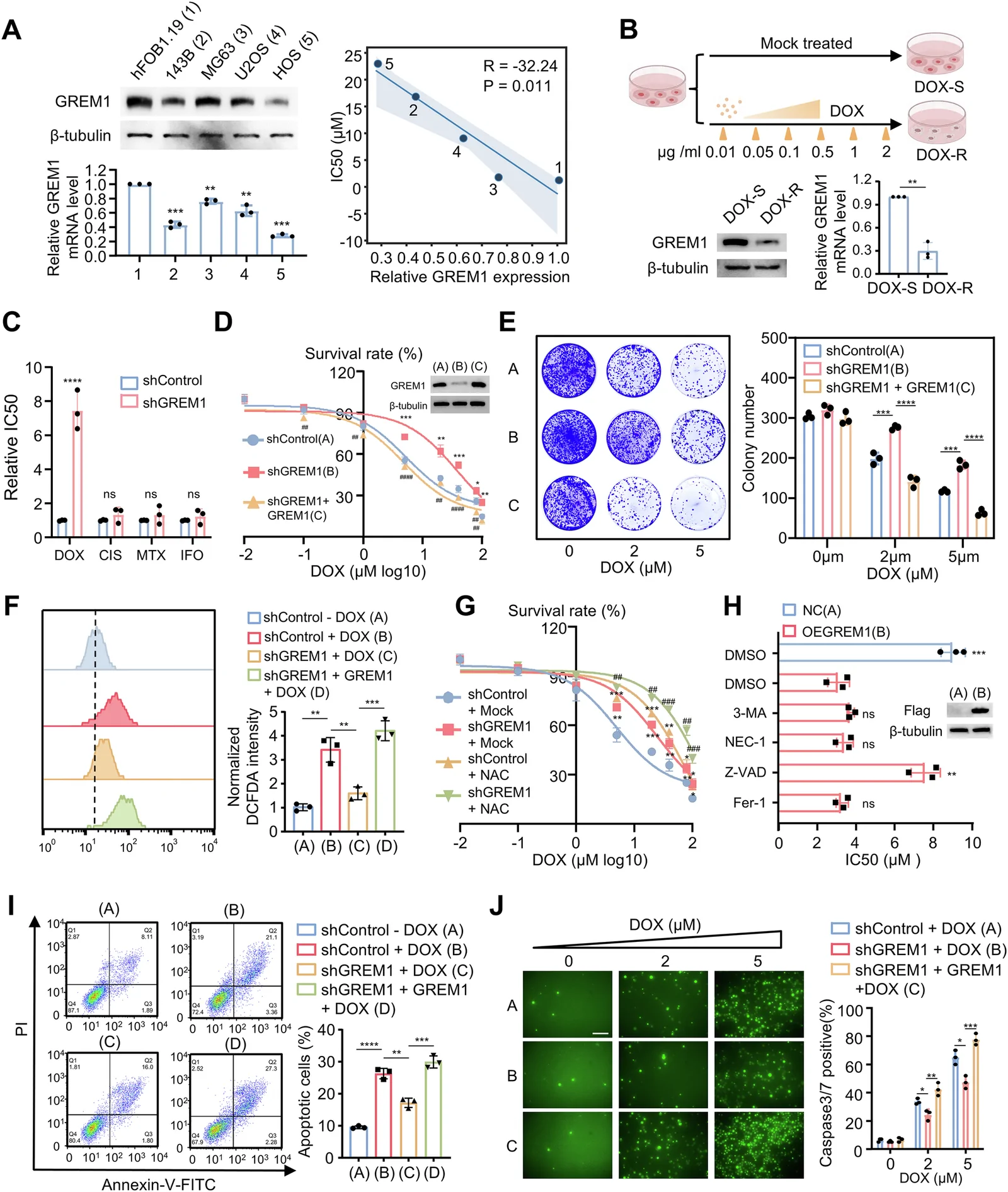
GREM1 enhances osteosarcoma sensitivity to DOX by promoting ROS-induced cell apoptosis. **A** GREM1 expression in hFOB1.19 and four osteosarcoma cell lines assessed by qRT-PCR and WB; DOX IC_50_ values for five lines were determined. Representative Western blots shown with β-Tubulin as a control. **B** Strategy for establishing DOX-resistant (DOX-R) MG63 cells; GREM1 expression in DOX-sensitive (DOX-S) and DOX-R MG63 cells analyzed by qRT-PCR and WB. **C** IC_50_ determination for four drugs in GREM1-knockdown U2OS cells. **D** GREM1 knockdown and re-expression in U2OS cells; cell viability after DOX treatment assessed using CCK-8 assay. **E** Colony formation assay used to evaluate survival and proliferation. **F** ROS levels measured by flow cytometry in treated U2OS cells. **G** Cell viability in shGREM1 or shControl U2OS cells exposed to DOX, with or without NAC (1 mM). **H** Cell viability in OEGREM1 or NC U2OS cells treated with DOX, with or without Z-VAD (40 μM), Fer-1 (1 μM), Nec-1 (10 μM), or 3-MA (5 mM). **I** Apoptosis rates in treated U2OS cells measured by flow cytometry. **J** Caspase-3/7 staining to assess apoptosis. Scale bar = 100 μm. Error bars represent SD; *p* values calculated by two-tailed unpaired *t*-test; *p < 0.05; **p < 0.01; ***p < 0.001; ****p < 0.0001; ^##^*p* < 0.01; ^###^*p* < 0.001. *n* = 3.

To define the role of GREM1 in the response of osteosarcoma cells to doxorubicin (DOX)-induced cell death, we examined GREM1 expression in four osteosarcoma cell lines (143B, MG63, U2OS, HOS) and a normal osteoblast line (hFOB1.19). qRT-PCR and WB both showed that GREM1 expression was significantly lower in osteosarcoma cell lines compared to hFOB1.19. We next determined the half-maximal inhibitory concentration (IC_50_) of DOX across the cell lines and observed a positive correlation between GREM1 expression and DOX sensitivity (Fig. 2A). Based on this, we selected U2OS and MG63—both with higher GREM1 levels—for knockdown experiments. Additionally, we established a DOX-resistant MG63 cell line and detected decreased GREM1 expression in these resistant cells (Fig. 2B). After constructing the GREM1 knockdown cell line, we added four first-line chemotherapy drugs for osteosarcoma, including DOX, to demonstrate that alterations in GREM1 can specifically affect DOX sensitivity (Fig. 2C). Knocking down GREM1 in osteosarcoma cells reduced their sensitivity to DOX, while restoring GREM1 expression resensitized the cells (Fig. 2D; Supplementary Fig. S1B). This effect was further confirmed by the colony formation assay (Fig. 2E; Supplementary Fig. S1C). GREM1 suppression also led to a drop in DOX-induced reactive oxygen species (ROS) levels, an effect reversed when GREM1 was re-expressed (Fig. 2F; Supplementary Fig. S1D). In line with this, the ROS scavenger N-acetylcysteine (NAC) significantly raised osteosarcoma cell resistance to DOX, indicating that GREM1 might act by promoting ROS accumulation (Fig. 2G; Supplementary Fig. S1E). To dissect the cell death pathway involved, we performed IC_50_ assays using pathway-specific inhibitors: ferrostatin-1 (Fer-1, ferroptosis), Z-VAD-FMK (Z-VAD, apoptosis), 3-methyladenine (3-MA, autophagy), and necrostatin-1 (Nec-1, necroptosis). Among these, only Z-VAD significantly rescued GREM1-induced cell death under DOX exposure (Fig. 2H; Supplementary Fig. S1F). Moreover, GREM1 overexpression clearly increased the rate of DOX-induced apoptosis (Fig. 2I and J; Supplementary Fig. S1G and S1H). Together, these results suggest that GREM1 enhances osteosarcoma cell sensitivity to DOX by driving apoptosis through elevated ROS production. Among them, hFOB1.19 cells are conditionally immortalized osteoblasts, and future validation in primary cells or models closer to physiological states would be a beneficial supplement.

### GREM1 exacerbates DOX-induced mitochondrial fission and dysfunction

**Fig. 3:**
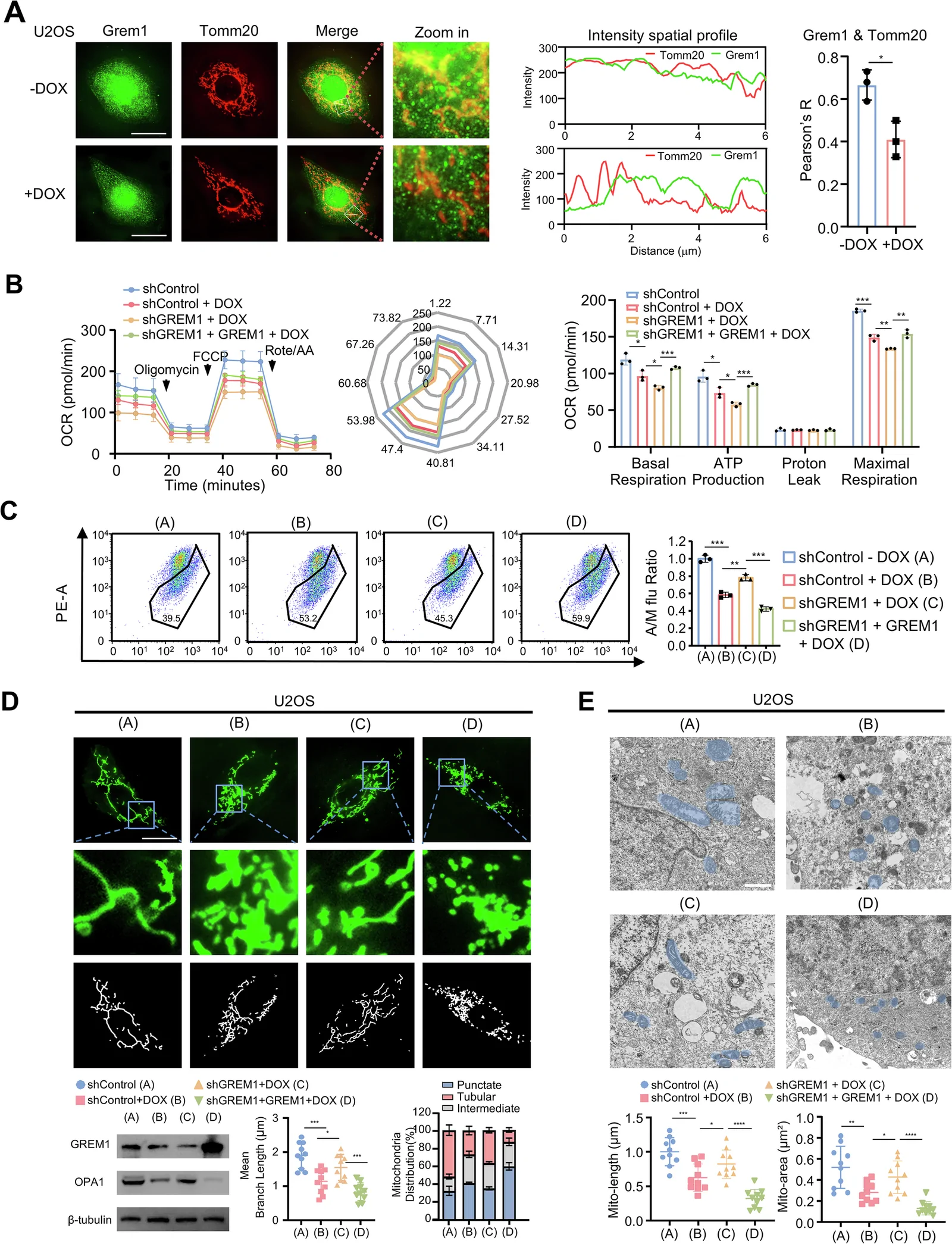
GREM1 exacerbates DOX-induced mitochondrial fission and dysfunction. **A** IF showing co-localization of TOMM20 (red)-labeled mitochondria and GREM1 (green) before and after DOX treatment. The left image displays fluorescence intensity distribution for TOMM20 and GREM1 along the same distance. The right histogram shows Pearson’s R coefficient quantifying co-localization. *n* = 3. Scale bar = 20 μm. **B** Oxygen consumption rates (OCR) were measured in shGREM1 and shControl U2OS cells. Bar graphs show basal respiration, ATP production, proton leak, and maximum respiration. *n* = 3. **C** MMP was analyzed using A (aggregate)/M (monomer) fluorescence ratios by flow cytometry. *n* = 3. **D** Mitochondrial fusion and fission were visualized using MitoTracker Green. Mean branch length was quantified with ImageJ, and mitochondrial forms were categorized into Punctate, Tubular, and Intermediate types. *n* = 10. **E** Transmission electron microscopy used to examine mitochondrial ultrastructure. *n* = 10. Error bars indicate SD; *p* values calculated by two-tailed unpaired *t*-test; *p < 0.05; **p < 0.01; ***p < 0.001; ****p < 0.0001.

Since mitochondria are the main source of endogenous ROS, we examined the influence of GREM1 on mitochondrial function. IF showed that GREM1 localizes in both the nucleus and cytoplasm. Following DOX treatment, its co-localization with mitochondria was reduced (Fig. 3A; Supplementary Fig. S2A). Additionally, oxygen consumption rate (OCR)—a key measure of mitochondrial oxidative respiration—was decreased (Fig. 3B). This reduction in OCR was more pronounced with GREM1 knockdown, while re-expression of GREM1 restored it (Fig. 3B; Supplementary Fig. S2B). These results suggest that GREM1 influences DOX resistance by affecting mitochondrial respiration.

Mitochondrial membrane potential (MMP) is crucial for maintaining oxidative phosphorylation and ATP synthesis. DOX exposure led to a decline in MMP, as shown by a reduced A (aggregate)/M (monomer) fluorescence ratio (Fig. 3C; Supplementary Fig. S2C). GREM1 knockdown alleviated the DOX-induced MMP reduction, whereas GREM1 overexpression reversed this protective effect. Mitochondria are dynamic organelles that continuously undergo division and fusion within the cell. In response to stimulation or injury, they alter their structure to accommodate the cell’s energy and physiological demands. The decrease in MMP levels, indication of mitochondrial damage induced by DOX and exacerbated by GREM1, led us to investigate the potential role of GREM1 in regulating mitochondrial morphology. Mitochondrial staining and transmission electron microscopy revealed fragmented mitochondria after DOX treatment. GREM1 depletion encouraged fusion, while its re-expression intensified mitochondrial fission (Fig. 3D and E; Supplementary Fig. S2D and S2E). Taken together, these findings indicate that GREM1 aggravates DOX-induced mitochondrial fission and dysfunction.

### C/EBPα serves as an upstream transcription factor of GREM1 and can reverse DOX resistance in osteosarcoma

**Fig. 4:**
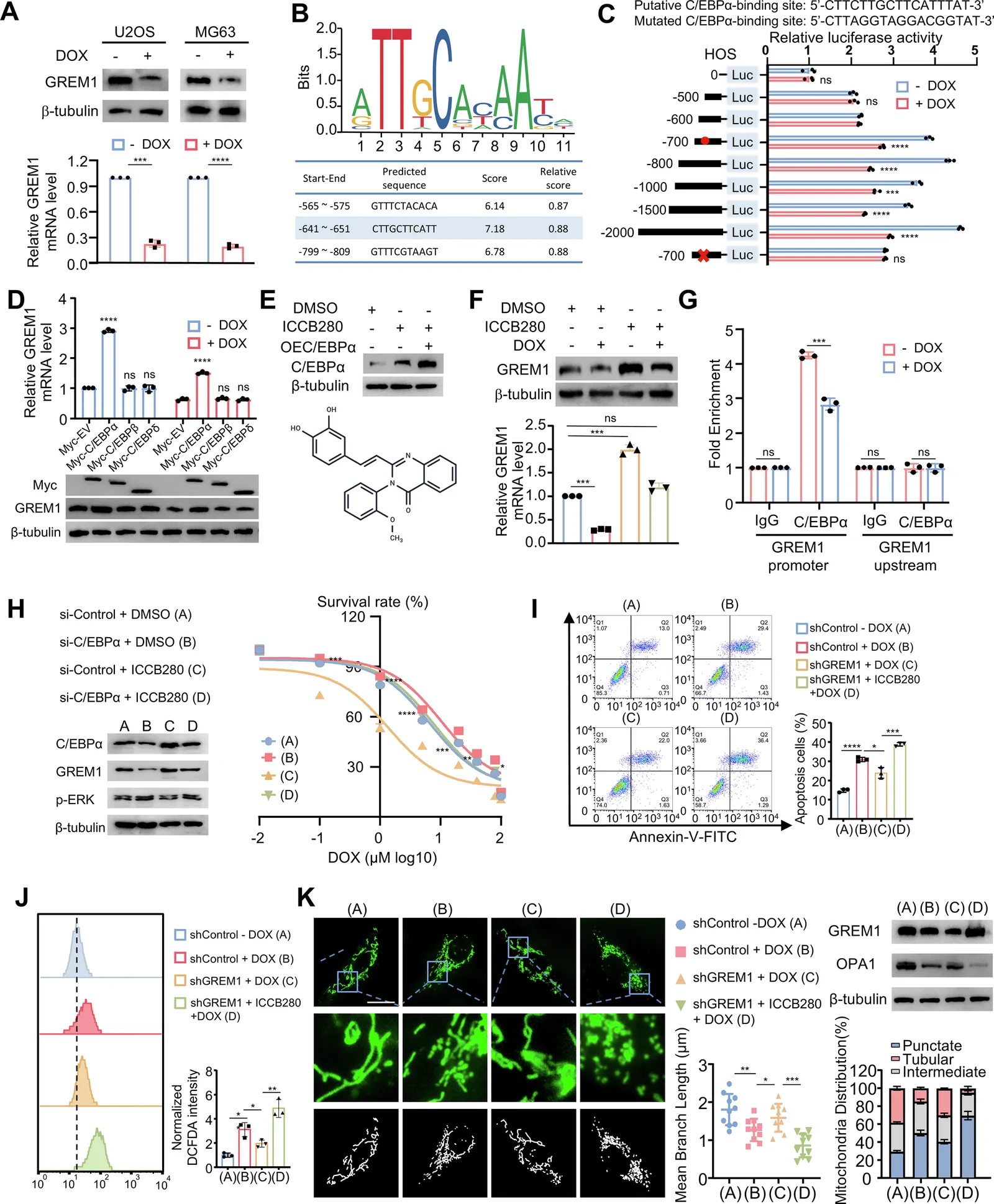
C/EBPα serves as an upstream transcription factor of GREM1 and can reverse DOX resistance in osteosarcoma. **A** GREM1 expression in osteosarcoma cells before and after DOX treatment measured by qRT-PCR and WB. Representative blots show GREM1 levels, with β-Tubulin as the internal control. *n* = 3. **B** Prediction of a conserved C/EBPα binding site within the GREM1 promoter using JASPAR (http://jaspar.genereg.net/). **C** Luciferase activity measured in U2OS cells transfected with a series of GREM1 promoter deletion constructs, with or without DOX treatment. The solid circle marks the predicted C/EBPα binding site; “X” indicates its mutated version. *n* = 3. **D** qRT-PCR of GREM1 expression in U2OS cells transfected with EV, C/EBPα, C/EBPβ, and C/EBPδ and treated with DOX. WBs show corresponding protein levels, with β-Tubulin as control. *n* = 3. **E** Chemical structure of ICCB280 and WB analysis of its induction effect on C/EBPα expression. ICCB280 = 10 μM. **F** GREM1 expression in osteosarcoma cells before and after ICCB280 treatment measured by qRT-PCR and WB. *n* = 3. **G** ChIP assay showing C/EBPα occupancy at the GREM1 promoter and upstream regions before and after DOX treatment. *n* = 3. **H** Cell viability of U2OS cells with knockdown of C/EBPα (si-C/EBPα) or exposure to ICCB280 were measured under treatment with a gradient concentration of DOX. Western blot was used to verify the expression levels of C/EBPα, GREM1, and p-ERK in U2OS cells. *n* = 3. **I** Apoptosis rate and (**J**) ROS levels in treated U2OS cells measured by flow cytometry. *n* = 3. **K** MitoTracker Green used to visualize mitochondrial structure; ImageJ software used to quantify average branch length and categorize mitochondrial forms into Punctate, Tubular, and Intermediate. *n* = 10. Error bars represent SD; *p* values from two-tailed unpaired *t*-test; *p < 0.05; **p < 0.01; ***p < 0.001; ****p < 0.0001.

To investigate upstream regulators of GREM1, we measured both transcriptional and protein levels of GREM1 in U2OS and MG63 cells under DOX treatment. GREM1 expression declined following exposure to DOX (Fig. 4A). We then examined GREM1 promoter activity and found it to be suppressed after DOX treatment. Utilizing a series of GREM1 promoter deletion reporter constructs, we identified a region between -700 and -600 bp that harbored DOX-responsive elements. As determined by bioinformatics analysis (http://tfbind.hgc.jp), C/EBPα seems to be a potent promoter. It was discovered that, mutation of the putative C/EBPα binding site within this region abolished its responsiveness to DOX (Fig. 4B and C). Among the C/EBP family, only C/EBPα consistently affected GREM1 expression under both untreated and DOX-treated conditions, suggesting its specificity as a transcriptional regulator (Fig. 4D). We selected ICCB280, a known C/EBPα agonist, for further study. Compared to direct overexpression, ICCB280 more effectively induced C/EBPα expression (Fig. 4E). ICCB280 also significantly upregulated GREM1 levels and reversed the DOX-induced downregulation of GREM1 (Fig. 4F). Chromatin immunoprecipitation (ChIP) confirmed that C/EBPα binds directly to the GREM1 promoter, but not to upstream regions, and this interaction was weakened after DOX treatment (Fig. 4G). In addition, we verified the targeting effect of ICCB280. The results showed that under C/EBPα-deficient conditions, the enhanced sensitivity to DOX mediated by ICCB280 was eliminated, indicating that ICCB280 targets and stimulates C/EBPα to exert its effect (Fig. 4H). As expected, ICCB280 enhanced DOX-induced cytotoxicity, increased ROS production, and promoted mitochondrial fission in osteosarcoma cells, all in a GREM1-dependent manner. These results indicate that C/EBPα regulates DOX sensitivity, mitochondrial fission, and ROS levels through regulation of GREM1 (Fig. 4I–K; Supplementary Fig. S3A–S3D).

### GREM1 enhances DOX doxorubicin sensitivity through the MAPK/ERK pathway

**Fig. 5:**
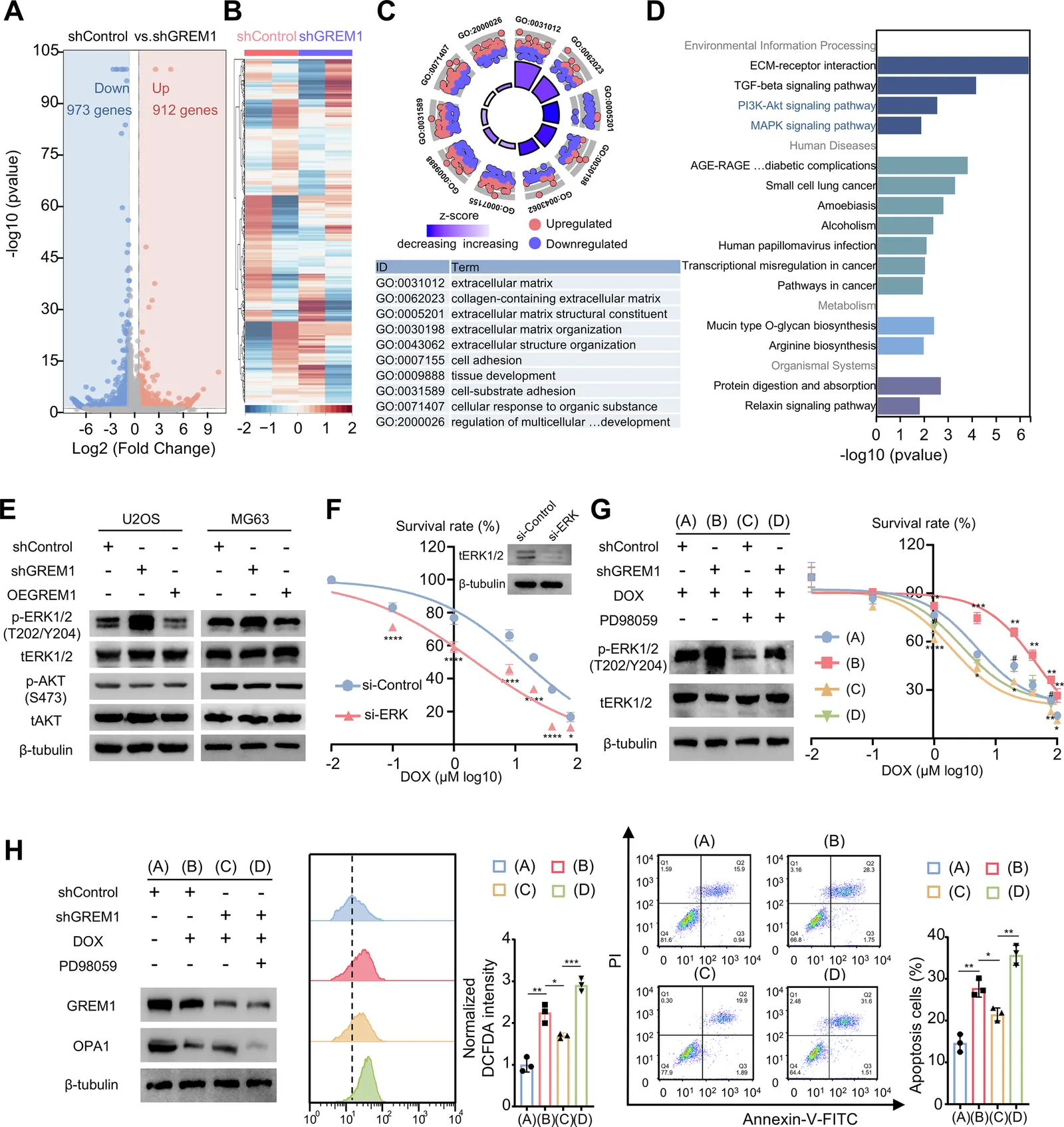
GREM1 enhances DOX sensitivity through the MAPK/ERK pathway. **A** Volcano plot and (**B**) heatmap showing differential gene expression between GREM1 knockdown (shGREM1) and control (shControl) U2OS cells. **C** GO and (**D**) KEGG enrichment analysis of the DEGs. **E** WB analysis of downstream signaling pathway changes following GREM1 knockdown (shGREM1) or overexpression (OEGREM1); β-Tubulin used as internal control. **F** Cell viability of U2OS cells with knockdown of ERK (si-ERK) was measured under treatment with a gradient concentration of DOX. Western blot was used to verify the expression levels of p-ERK in osteosarcoma cells. **G** WB and cell viability analysis of shControl and shGREM1 U2OS cells treated with DOX alone or in combination with PD98059. PD98059 = 10 μM. **H** ROS levels and apoptosis rates measured by flow cytometry in treated U2OS cells, and OPA1 expression analyzed by WB. Error bars represent SD; *p* values calculated by two-tailed unpaired *t*-test; *p < 0.05; **p < 0.01; ***p < 0.001; ****p < 0.0001; ^#^*p* < 0.05. *n* = 3.

To investigate how GREM1 affects DOX sensitivity, we performed transcriptome sequencing on U2OS cells following GREM1 knockdown. A total of 1885 genes showed significantly differential expression, with 912 upregulated and 973 downregulated in the knockdown group (Fig. 5A and B) (GEO accession: GSE282643). GO enrichment analysis revealed that these genes were mainly involved in processes such as cell adhesion, extracellular matrix organization, and tissue development, indicating that GREM1 plays a role in maintaining osteosarcoma cell homeostasis (Fig. 5C). KEGG pathway analysis identified changes in several cancer-related pathways, notably MAPK and PI3K-AKT, suggesting that GREM1 may exert its effects through these signaling routes (Fig. 5D). To test this, we assessed pathway activity following GREM1 knockdown or overexpression. Results showed that GREM1 primarily influenced the ERK arm of the MAPK pathway, while the AKT pathway remained largely unaffected (Fig. 5E). In addition, after knocking down ERK, the sensitivity of osteosarcoma cells to DOX can be enhanced, confirming that ERK is a downstream effector in the pathway (Fig. 5F). Treating cells with the ERK inhibitor PD98059 increased the DOX sensitivity of both GREM1-knockdown and DOX-resistant osteosarcoma cells and reduced OPA1 expression (Fig. 5G–H; Supplementary Fig. S4A–S4D).

### Manipulation of the C/EBPα/GREM1/p-ERK axis enhances the efficacy of DOX in vivo

**Fig. 6:**
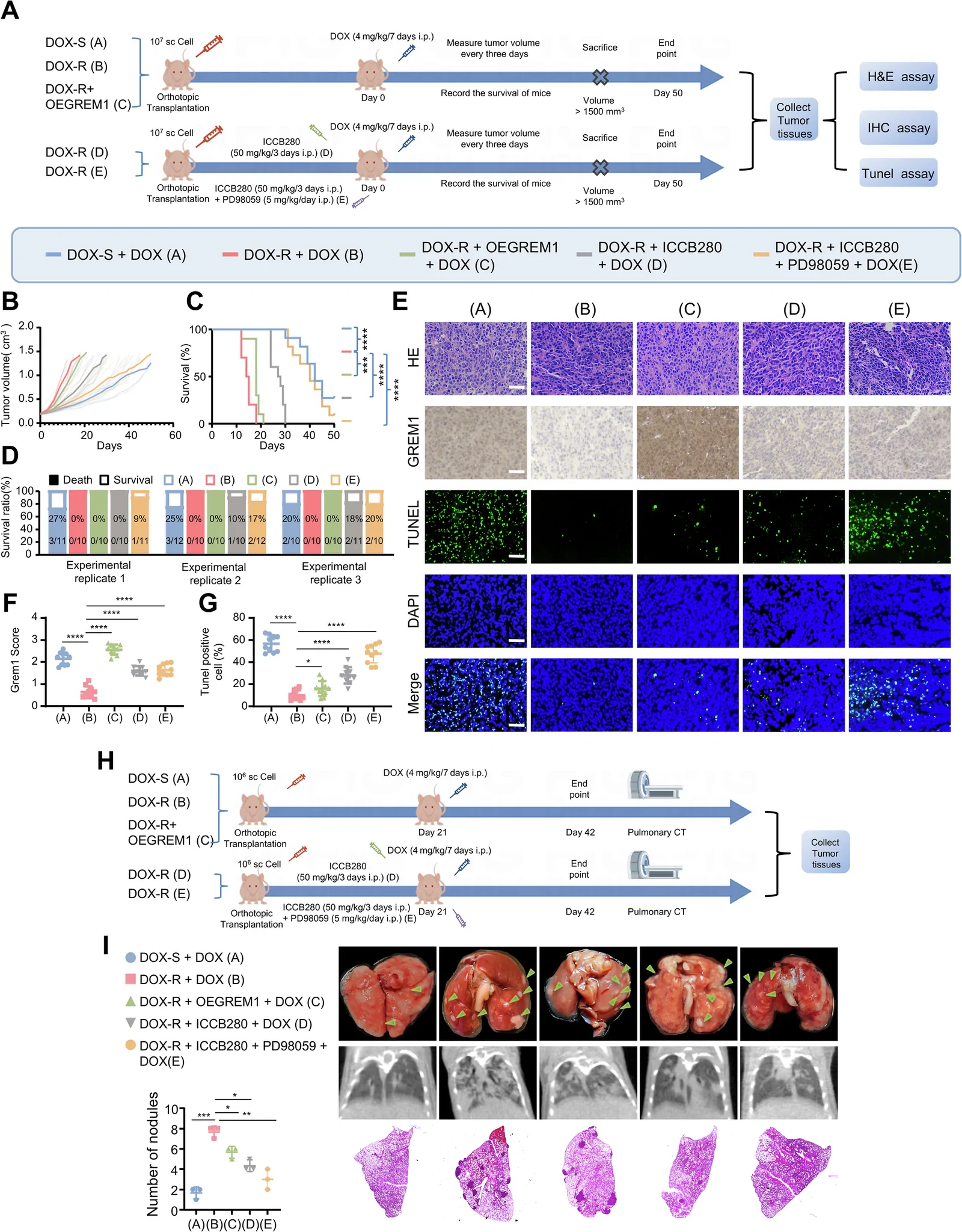
Regulating the C/EBPα/GREM1/p-ERK axis promotes the therapeutic effect of DOX and suppresses osteosarcoma lung metastasis. **A** Schematic of the in vivo study. Nude mice were subcutaneously injected with DOX-R, DOX-S, or DOX-R + OEGREM1 MG63 cells. When tumor volume exceeded 200 mm^3^, DOX (4 mg/kg every 7 days) was administered intraperitoneally, alone or in combination with ICCB280 (50 mg/kg every 3 days) or ICCB280 plus PD98059 (5 mg/kg/day). Mice were euthanized when tumors exceeded 1500 mm^3^. The study ended on day 50, with mice bearing tumors smaller than 1500 mm^3^ considered survivors. Each group contains 10-12 nude mice. **B** Tumor volume was recorded every three days. **C** Survival curves were plotted for each group. **D** Bar chart showing survival rates across three independent experiments. **E**–**G** Representative H&E, IHC, and TUNEL staining of tumor sections. Scatter plots show apoptotic cell ratios and GREM1 expression. Scale bar = 50 μm. **H** Using the same grouping and treatment as in (**A**), lung CT scans were performed on day 42. Lungs were collected after scanning. Each group contains 3 nude mice. **I** Representative gross lung images, CT scans, and H&E-stained sections. Scatter plots show the number of visible lung tumor nodules. Error bars indicate SD; *p* values from two-tailed unpaired *t*-test; *p < 0.05; ***p < 0.001; ****p < 0.0001.

ICCB280 is an effective C/EBPα inducer that activates C/EBPα and affects its downstream targets. PD98059 is a selective MEK inhibitor that can effectively block the phosphorylation of ERK. In previous studies, we found that both compounds can increase the sensitivity of osteosarcoma cells to DOX in vitro, we next examined whether these compounds could improve DOX efficacy in vivo. DOX-sensitive (DOX-S), DOX-resistant (DOX-R), and GREM1-overexpressing DOX-R (DOX-R + OEGREM1) MG63 cells were subcutaneously injected into nude mice. Once tumors were palpable, DOX was administered intraperitoneally at 4 mg/kg every seven days, either alone or in combination with ICCB280 or ICCB280 plus PD98059 (Fig. 6A). As shown in Fig. 6B, tumor growth in the DOX-S group slowed significantly, with three mice surviving up to 50 days. In contrast, DOX-R tumors grew rapidly, resulting in shorter survival. GREM1 overexpression moderately restored DOX sensitivity in the DOX-R tumors. Notably, ICCB280 treatment significantly improved DOX’s antitumor effect. The combined administration of ICCB280 and PD98059 further improved DOX efficacy, achieving outcomes similar to those seen in DOX-S tumors (Fig. 6C and D). Apoptosis analysis revealed a significantly higher proportion of apoptotic cells in tumors treated with the DOX + ICCB280 + PD98059 combination (Fig. 6E–G). These results indicate that targeting the GREM1 axis can amplify the therapeutic effect of DOX in vivo. Given the high rate of lung metastasis in osteosarcoma, we also evaluated whether ICCB280 and PD98059 could improve the efficacy of systemic DOX in a lung metastasis model (Fig. 6H). Consistent with results from subcutaneous tumors, ICCB280 and PD98059 significantly increased DOX effectiveness against lung metastases in the in vivo osteosarcoma model (Fig. 6I).

### The regulation of C/EBPα/GREM1/p-ERK axis is related to the clinical and pathological characteristics of osteosarcoma patients

**Fig. 7:**
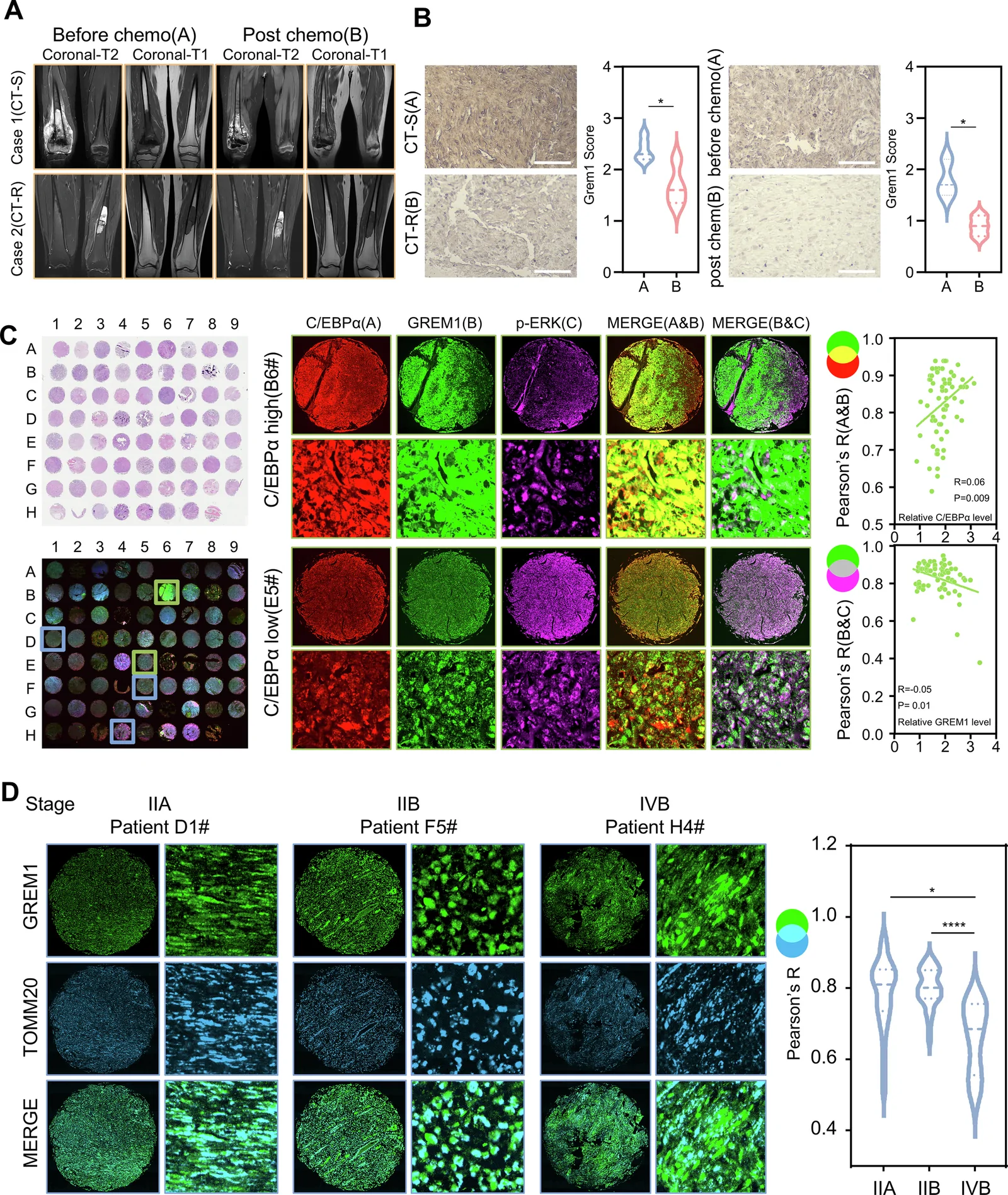
Regulation of the C/EBPα/GREM1/p-ERK axis is linked to clinical and pathological features of osteosarcoma patients. **A** MRI comparison of two patient groups before and after chemotherapy. **B** Immunohistochemistry (IHC) assessing GREM1 expression in chemotherapy-sensitive (CT-S) and chemotherapy-resistant (CT-R) patients. Scale bar = 100 μm. **C** Correlation between C/EBPα, GREM1 and p-ERK expression based on tissue microarray analysis from 70 osteosarcoma samples and one normal bone sample located at site H8. *n* = 70. **D** Patients were grouped by clinical stage, and co-localization of mitochondria labeled with GREM1 and TOMM20 was analyzed across different tumor stages. *n* = 70. Error bars indicate SD; *p* values calculated using two-tailed unpaired *t*-test; *p < 0.05; ***p < 0.001; ****p < 0.0001.

To evaluate the clinical relevance of our findings, we collected osteosarcoma tissue samples from nine patients, including both pre- and post-chemotherapy conditions and cases with either sensitivity or resistance to treatment. In chemotherapy-sensitive patients, magnetic resonance imaging (MRI) revealed reduced tumor-associated edema and increased necrotic areas following chemotherapy (Fig. 7A). Immunohistochemistry showed that GREM1 expression was higher in chemotherapy-sensitive patients compared to resistant ones, with a noticeable reduction in GREM1 levels after chemotherapy (Fig. 7B). To further examine the relationship between GREM1, C/EBPα, and p-ERK, we analyzed tissue microarrays from 70 osteosarcoma cases. As C/EBPα expression increased, its co-localization with GREM1 also rose, while co-localization of GREM1 with p-ERK decreased, based on Pearson’s correlation analysis (Fig. 7C). We also found that as tumor grade advanced, the co-localization of GREM1 with TOMM20 decreased, suggesting a decrease in mitochondrial localization of GREM1. This observation hints that the mitochondrial role of GREM1 may contribute to osteosarcoma heterogeneity (Fig. 7D). Finally, we extracted a portion of specimens from osteosarcoma surgery for cell culture to verify the presence of the C/EBPα/GREM1/p-ERK signaling axis in primary osteosarcoma cells (Supplementary Fig. S4E). Altogether, these results support the clinical significance of GREM1 in osteosarcoma chemotherapy response and show that the C/EBPα/GREM1/p-ERK axis is linked to chemotherapy outcomes in osteosarcoma.

## Discussion

Doxorubicin remains a key chemotherapeutic agent for treating osteosarcoma. Its cytotoxicity stems from multiple intracellular actions, including ROS generation, DNA-adduct formation, inhibition of topoisomerase II, histone displacement, disruption of Ca^2+^ and iron homeostasis, and ceramide accumulation [12]. However, resistance to DOX continues to pose a major obstacle in osteosarcoma therapy. This study sought to overcome that resistance and improve the therapeutic impact of DOX in osteosarcoma. We focused on the gene GREM1, which appears to play a role in enhancing chemotherapeutic efficacy in osteosarcoma patients undergoing treatment with DOX-containing regimens. Our in vitro experiments showed that GREM1 overexpression increases osteosarcoma cell sensitivity to DOX by driving apoptosis through elevated ROS production. At the mechanistic level, GREM1 regulates mitochondrial fission via the MAPK/ERK pathway, and its transcription is activated by C/EBPα. Based on this, the combined use of a C/EBPα agonist (ICCB280) and an ERK pathway inhibitor (PD98059) with DOX can effectively re-sensitize resistant osteosarcoma tumors to treatment.

GREM1, a member of the cysteine-rich DAN family of secreted proteins, is known to bind bone morphogenetic proteins (BMPs) and inhibit their activity [13]. It plays a vital role in developmental regulation and the maintenance of tissue function [14]. Since its identification, GREM1 has been widely studied across multiple disease contexts, including diabetic nephropathy, idiopathic pulmonary fibrosis, and pulmonary hypertension. In oncology, GREM1 displays complex, context-dependent functions. It has been linked to cancer progression in gastric, breast, and glioblastoma cancers [15–17], where elevated expression often corresponds with more aggressive tumor behavior and worse prognosis. Conversely, in cancers such as renal cell carcinoma and pancreatic neuroendocrine tumors, GREM1 functions as a tumor suppressor and is associated with improved survival [18, 19]. A 2022 study published in *Nature* highlighted GREM1’s role in mesenchymal pancreatic ductal adenocarcinoma (PDAC), where its upregulation suppressed key epithelial-mesenchymal transition transcription factors including Snai1 and Snai2, ultimately reducing PDAC malignancy and metastatic potential [20]. In osteosarcoma, GREM1 was previously reported to act as a tumor suppressor by inhibiting cell proliferation and invasion [21]. Our findings build on this, showing that GREM1 is also critical in increasing the sensitivity of osteosarcoma cells to the chemotherapeutic agent DOX.

The transcription factor CCAAT/enhancer binding protein α (C/EBPα) serves as a pivotal regulator of lineage-specific gene expression and is a primary regulatory molecule in the terminal differentiation of various cell types, including granulocytes. It is mainly studied in leukemia and has been found to have the ability to regulate tumor proliferation in other solid tumors such as liver cancer and cervical cancer. Recent evidence suggests C/EBPα interacts with Runx3 in OS, where it can inhibit the oncogenic Runx3-Myc pathway. The expression changes of C/EBPα also have a regulatory effect on drug sensitivity, such as the LSD1 inhibitors GSK-LSD1 and Veneto. However, the role of C/EBPα in the sensitivity of osteosarcoma chemotherapy has not been reported yet. Various agonists and inhibitors of C/EBPα are also being studied, among which MTL-CEBPA has significant anti-tumor effects in liver cancer. The ICCB280 we used is a potent C/EBPα inducer that activates this transcription factor and regulates its downstream targets, leading to myeloid differentiation of leukemia cells in terms of morphology and function. The short half-life is a key factor limiting the clinical application potential of small molecule agonists or inhibitors. However, there are currently no reports on the pharmacokinetics of ICCB280. Our study demonstrates that C/EBPα significantly enhances doxorubicin’s antitumor effects both in vitro and in vivo through GREM1 induction, highlighting its clinical potential for overcoming chemoresistance in osteosarcoma treatment.

Mitochondria play a critical role in responding to cellular stress, adapting through processes such as fusion, fission, transport, and transfer. These mechanisms are also key to adjusting mitochondrial metabolism under drug pressure, contributing to resistance [22]. In non-small cell lung cancer, increased mitochondrial fragmentation via PIM inhibition sensitizes cells to paclitaxel [23], whereas mitochondrial fusion driven by N-Myc overexpression promotes resistance to cisplatin [24]. Our study is the first to show that GREM1 regulates mitochondrial fission-fusion balance and, as a result, strengthens the cytotoxic effect of DOX in osteosarcoma. This function positions GREM1 as a promising therapeutic target for treating DOX-resistant osteosarcoma. In future research, we can introduce fission inhibitors or fusion inhibitors that directly act on mitochondria to verify the accuracy of our results and explore the killing effect of drug combination on osteosarcoma.

Osteosarcoma typically shows an initial response to chemotherapy, but it often develops resistance quickly, which remains a major cause of treatment failure. Targeting the molecular mechanisms behind chemotherapy action, or proteins and signaling pathways that are abnormally expressed in osteosarcoma, may offer paths to overcome this resistance. For example, the PARP1 inhibitor Olaparib, which blocks DNA repair following chemotherapy-induced damage, has been shown to work synergistically with DOX to induce apoptosis in osteosarcoma cells [25]. This compound has already been explored in clinical trials. Another target, IGF-1R, a tyrosine kinase receptor often overexpressed in osteosarcoma, is linked to tumor metastasis and poor prognosis. Its inhibition with AG1024 has been reported to increase osteosarcoma cell sensitivity to DOX [26]. This study screened and validated the role of MAPK/ERK as a downstream effector of GREM1 through RNA-Seq, but other members of the MAPK pathway, such as p38 and JNK branches, still need to be explored. This study suggests that targeting the C/EBPα/GREM1/p-ERK axis represents a promising strategy to overcome DOX resistance. Although PD98059 served as a tool compound to validate the concept of targeting the ERK pathway in this context, its inherent pharmacological properties limit direct clinical translation. Future research should focus on developing clinical-grade ERK inhibitors with enhanced selectivity and favorable pharmacokinetic profiles (such as certain compounds currently under clinical investigation), or on exploring alternative approaches to intervene in this axis, to advance this strategy toward clinical therapeutic application.

Our study focused on GREM1, a gene that influences mitochondrial morphology and ROS production during chemotherapy. We also identified C/EBPα as an upstream transcription factor and ERK signaling as a downstream pathway in this axis. For the first time, the C/EBPα agonist ICCB280 and the ERK inhibitor PD98059 are shown to strengthen the cytotoxic effect of DOX and potentially reverse resistance in osteosarcoma. These findings suggest that ICCB280 and PD98059 may serve as candidate drugs to improve the sensitivity of untreated osteosarcoma and re-sensitize DOX-resistant osteosarcoma to chemotherapy.

## Supplementary information

Supplementary Figure S1

Supplementary Figure S2

Supplementary Figure S3

Supplementary Figure S4

Original Data

## Data Availability

The datasets generated and analysed during the current study are available in the [GEO] repository, [https://www.ncbi.nlm.nih.gov/geo/query/acc.cgi?acc=GSE282643].
