# Supplementary material for: C/EBP alpha agonist ICCB280 overcomes doxorubicin resistance in osteosarcoma through mitochondrial dynamics-dependent GREM1-MAPK activation
Source: Cell Death Dis. 2026 Jun 25;17(1):783. doi: 10.1038/s41419-026-08980-y (PMC13554245; doi:10.1038/s41419-026-08980-y)
Supplement: Supplementary file 1 [file 41419_2026_8980_MOESM5_ESM.pdf]

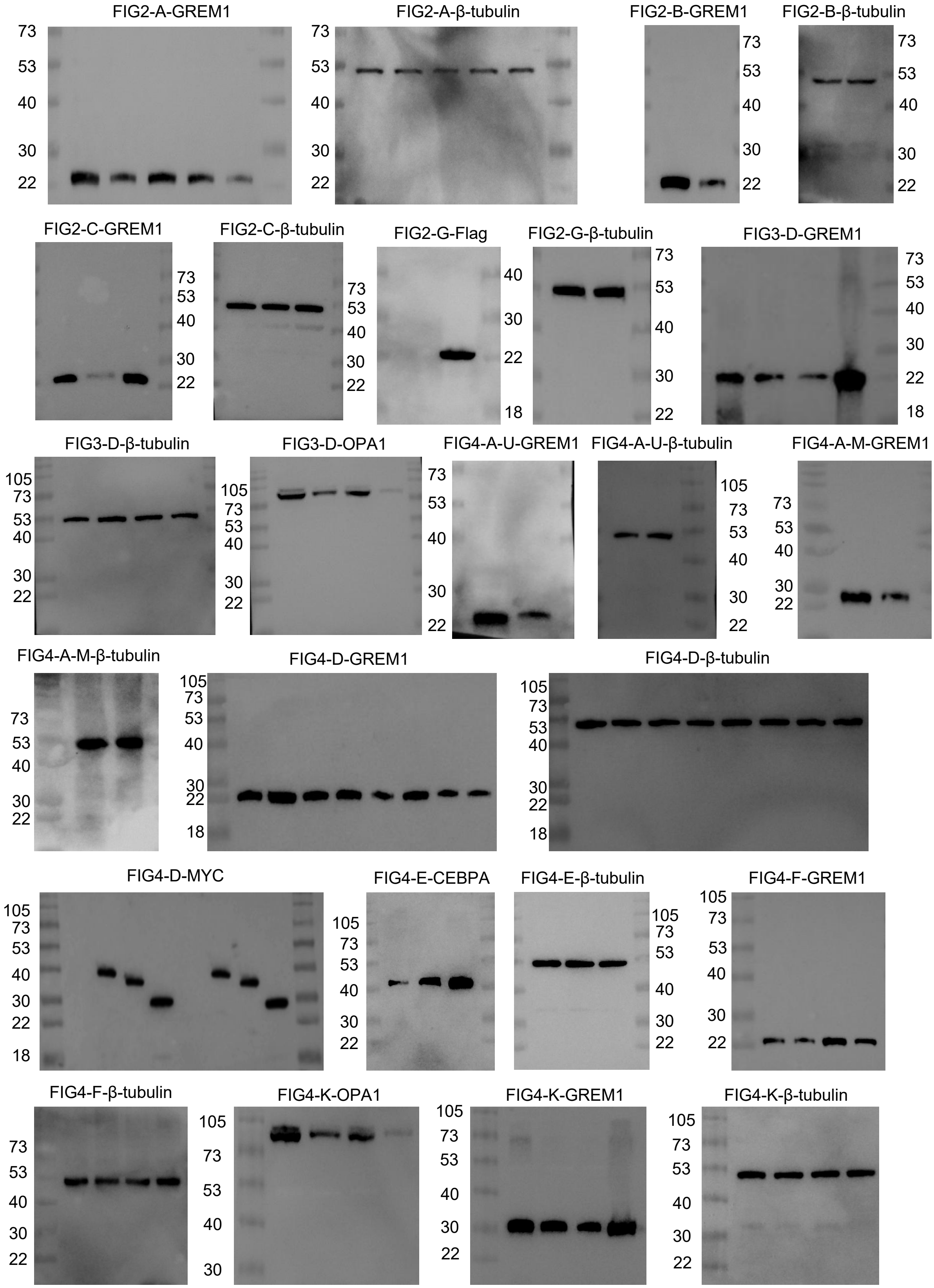
FIG2-A-GREM1
73 53 40 30 22
FIG2-A-β-tubulin
73 53 40 30 22
FIG2-B-GREM1
73 53 40 30 22
FIG2-B-β-tubulin
73 53 40 30 22
FIG2-C-GREM1
73 53 40 30 22
FIG2-C-β-tubulin
73 53 40 30 22
FIG2-G-Flag
40 30 22 18
FIG2-G-β-tubulin
73 53 40 30 22
FIG3-D-GREM1
73 53 40 30 22 18
FIG3-D-β-tubulin
105 73 53 40 30 22
FIG3-D-OPA1
105 73 53 40 30 22
FIG4-A-U-GREM1
73 53 40 30 22
FIG4-A-U-β-tubulin
105 73 53 40 30 22
FIG4-A-M-GREM1
73 53 40 30 22
FIG4-A-M-β-tubulin
73 53 40 30 22
FIG4-D-GREM1
105 73 53 40 30 22 18
FIG4-D-β-tubulin
105 73 53 40 30 22 18
FIG4-D-MYC
105 73 53 40 30 22 18
FIG4-E-CEBPA
105 73 53 40 30 22
FIG4-E-β-tubulin
105 73 53 40 30 22
FIG4-F-GREM1
105 73 53 40 30 22
FIG4-F-β-tubulin
73 53 40 30 22
FIG4-K-OPA1
105 73 53 40 30
FIG4-K-GREM1
105 73 53 40 30 22
FIG4-K-β-tubulin
105 73 53 40 30 22

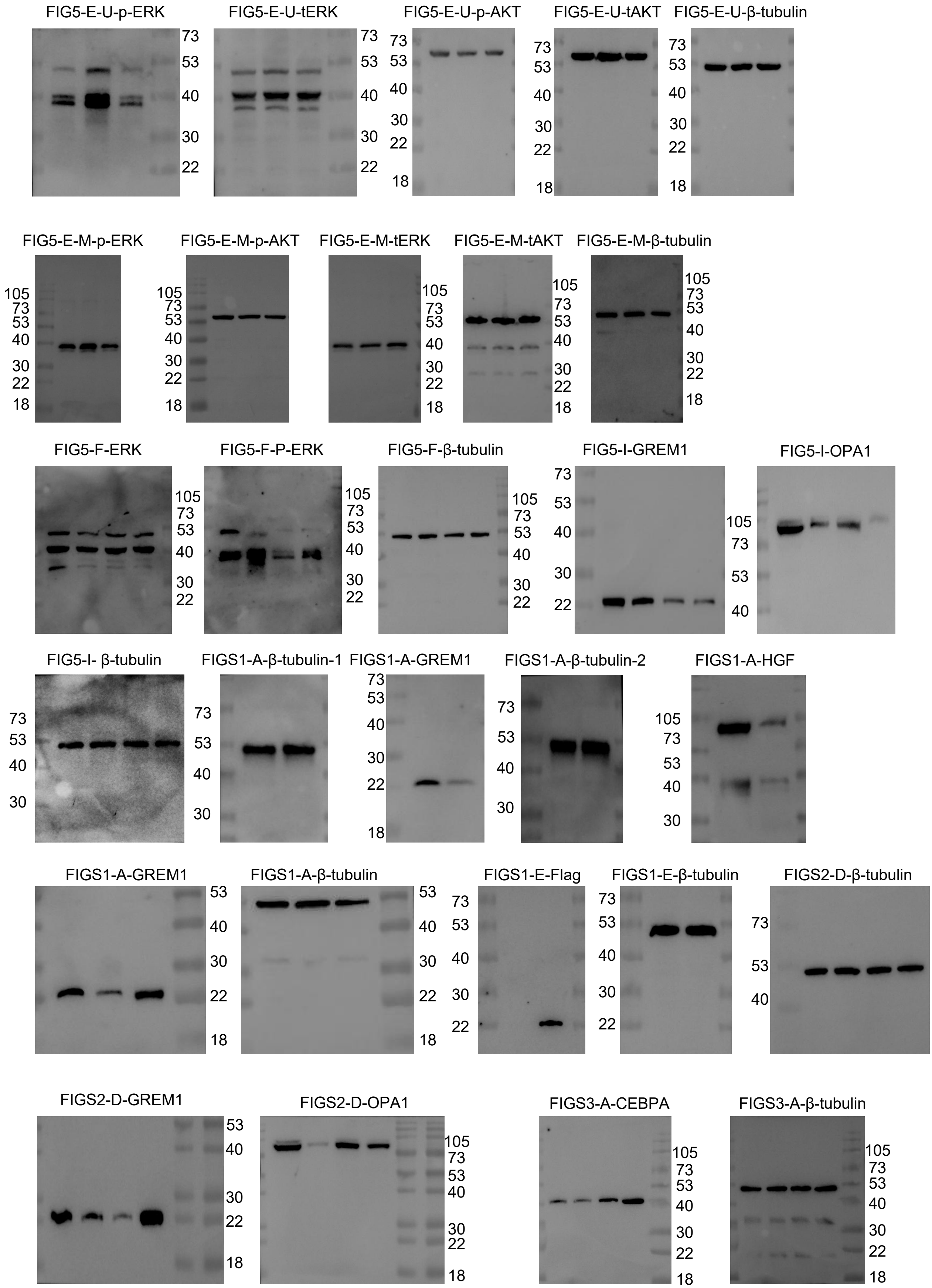
FIG5-E-U-p-ERK
73 53 40 30 22
FIG5-E-U-tERK
73 53 40 30 22
FIG5-E-U-p-AKT
73 53 40 30 22 18
FIG5-E-U-tAKT
73 53 40 30 22 18
FIG5-E-U-β-tubulin
73 53 40 30 22 18
FIG5-E-M-p-ERK
105 73 53 40 30 22 18
FIG5-E-M-p-AKT
105 73 53 40 30 22 18
FIG5-E-M-tERK
105 73 53 40 30 22 18
FIG5-E-M-tAKT
105 73 53 40 30 22 18
FIG5-E-M-β-tubulin
105 73 53 40 30 22 18
FIG5-F-ERK
105 73 53 40 30 22
FIG5-F-P-ERK
105 73 53 40 30 22
FIG5-F-β-tubulin
105 73 53 40 30 22
FIG5-I-GREM1
73 53 40 30 22
FIG5-I-OPA1
105 73 53 40
FIG5-I- β-tubulin
73 53 40 30
FIGS1-A-β-tubulin-1
73 53 40 30
FIGS1-A-GREM1
73 53 40 30 22 18
FIGS1-A-β-tubulin-2
73 53 40 30
FIGS1-A-HGF
105 73 53 40 30
FIGS1-A-GREM1
53 40 30 22 18
FIGS1-A-β-tubulin
53 40 30 22 18
FIGS1-E-Flag
73 53 40 30 22
FIGS1-E-β-tubulin
73 53 40 30 22
FIGS2-D-β-tubulin
73 53 40
FIGS2-D-GREM1
53 40 30 22 18
FIGS2-D-OPA1
105 73 53 40 30 22 18
FIGS3-A-CEBPA
105 73 53 40 30 22 18
FIGS3-A-β-tubulin
105 73 53 40 30 22 18

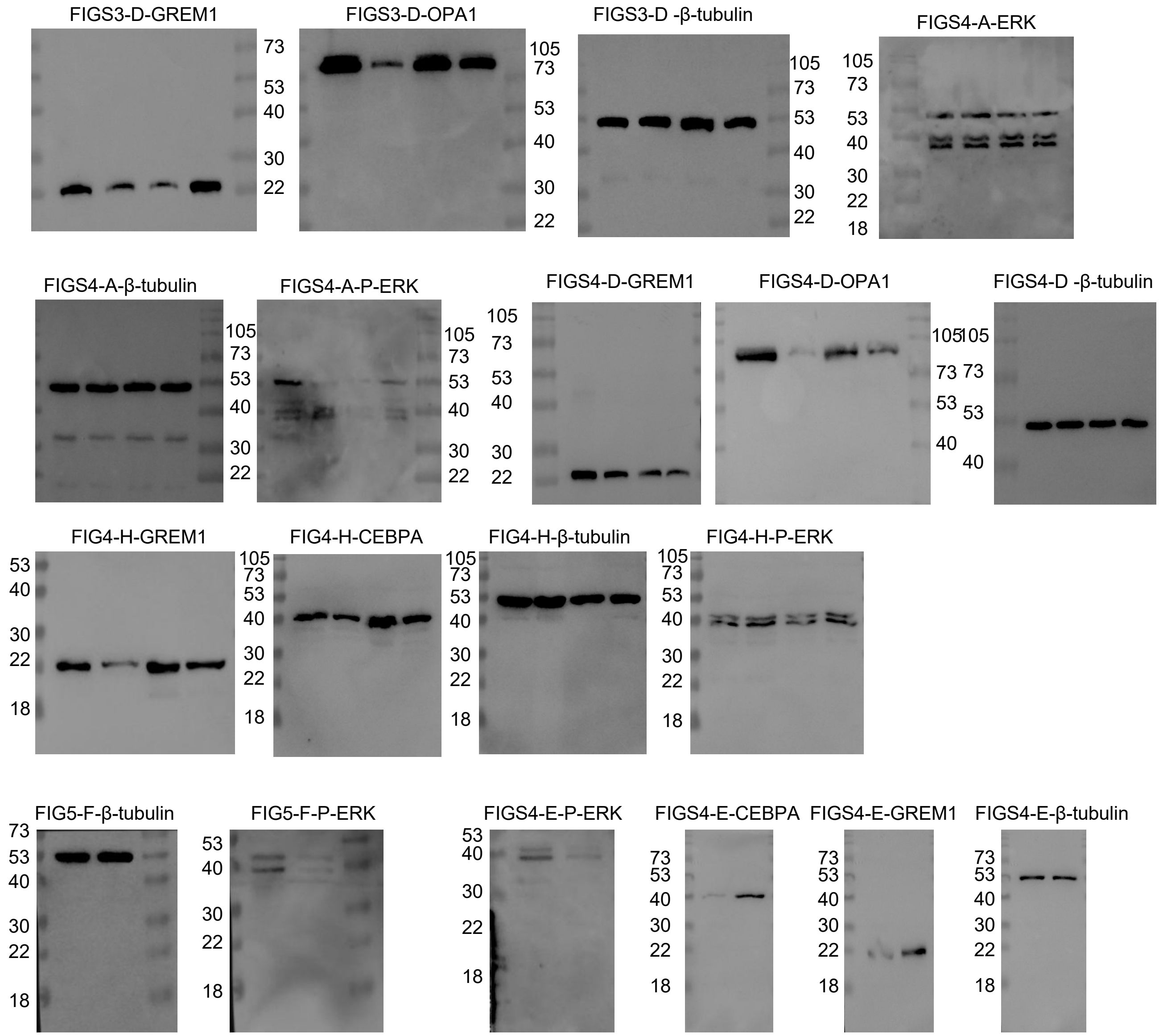

FIGS3-D-GREM1
73
53
40
30
22
FIGS3-D-OPA1
105
73
53
40
30
22
FIGS3-D -β-tubulin
105
73
53
40
30
22
FIGS4-A-ERK
105
73
53
40
30
22
18
FIGS4-A-β-tubulin
105
73
53
40
30
22
FIGS4-A-P-ERK
105
73
53
40
30
22
FIGS4-D-GREM1
105
73
53
40
30
22
FIGS4-D-OPA1
105
73
53
40
FIGS4-D -β-tubulin
105
73
53
40
FIG4-H-GREM1
53
40
30
22
18
FIG4-H-CEBPA
105
73
53
40
30
22
18
FIG4-H-β-tubulin
105
73
53
40
30
22
18
FIG4-H-P-ERK
105
73
53
40
30
22
18
FIG5-F-β-tubulin
73
53
40
30
22
18
FIG5-F-P-ERK
53
40
30
22
18
FIGS4-E-P-ERK
53
40
30
22
18
FIGS4-E-CEBPA
73
53
40
30
22
18
FIGS4-E-GREM1
73
53
40
30
22
18
FIGS4-E-β-tubulin
73
53
40
30
22
18
